# Field-Based Estimates of Global Warming Potential in Bioenergy Systems of Hawaii: Crop Choice and Deficit Irrigation

**DOI:** 10.1371/journal.pone.0168510

**Published:** 2017-01-04

**Authors:** Meghan N. Pawlowski, Susan E. Crow, Manyowa N. Meki, James R. Kiniry, Andrew D. Taylor, Richard Ogoshi, Adel Youkhana, Mae Nakahata

**Affiliations:** 1 Department of Natural Resources and Environmental Management, University of Hawaii Manoa, Honolulu, Hawaii, United States of America; 2 Texas A&M AgriLife Blackland Research and Extension Center, Temple, Texas, United States of America; 3 United States Department of Agriculture-Agricultural Research Service Grassland Soil and Water Research Laboratory, Temple, Texas, United States of America; 4 Department of Biology, University of Hawaii Manoa, Honolulu, Hawaii, United States of America; 5 Department of Tropical Plant and Soil Sciences, University of Hawaii Manoa, Honolulu, Hawaii, United States of America; 6 Hawaiian Commercial & Sugar, Puunene, Hawaii, United States of America; Pacific Northwest National Laboratory, UNITED STATES

## Abstract

Replacing fossil fuel with biofuel is environmentally viable from a climate change perspective only if the net greenhouse gas (GHG) footprint of the system is reduced. The effects of replacing annual arable crops with perennial bioenergy feedstocks on net GHG production and soil carbon (C) stock are critical to the system-level balance. Here, we compared GHG flux, crop yield, root biomass, and soil C stock under two potential tropical, perennial grass biofuel feedstocks: conventional sugarcane and ratoon-harvested, zero-tillage napiergrass. Evaluations were conducted at two irrigation levels, 100% of plantation application and at a 50% deficit. Peaks and troughs of GHG emission followed agronomic events such as ratoon harvest of napiergrass and fertilization. Yet, net GHG flux was dominated by carbon dioxide (CO_2_), as methane was oxidized and nitrous oxide (N_2_O) emission was very low even following fertilization. High N_2_O fluxes that frequently negate other greenhouse gas benefits that come from replacing fossil fuels with agronomic forms of bioenergy were mitigated by efficient water and fertilizer management, including direct injection of fertilizer into buried irrigation lines. From soil intensively cultivated for a century in sugarcane, soil C stock and root biomass increased rapidly following cultivation in grasses selected for robust root systems and drought tolerance. The net soil C increase over the two-year crop cycle was three-fold greater than the annualized soil surface CO_2_ flux. Deficit irrigation reduced yield, but increased soil C accumulation as proportionately more photosynthetic resources were allocated belowground. In the first two years of cultivation napiergrass did not increase net greenhouse warming potential (GWP) compared to sugarcane, and has the advantage of multiple ratoon harvests per year and less negative effects of deficit irrigation to yield.

## Introduction

Renewable energy is of growing domestic and global interest due to the depletion of fossil fuel reserves and concerns over energy security and climate change. Biofuels generated from agricultural crops are a favorable substitute for conventional fuel sources. However, if inappropriately managed, the production of biofuel feedstocks could be a net contributor to greenhouse gas (GHG) emissions [1]. In Hawaii, large-scale sugarcane (*Saccharum officinarum* L.) production was an important industry for more than a century, but in recent decades, there has been a drastic decline in production due to a number of factors, among which are low sugar prices, high labor costs, in particular, against competition from low cost foreign producers. In addition to this decline, concerns over local energy security, rising fuel costs, and competition for water resources have spurred interest in shifting from sugarcane production to select candidate bioenergy crops that optimize water and nutrient use efficiency, while also offering the potential to mitigate GHG emissions.

Carbon dioxide (CO_2_), methane (CH_4_) and nitrous oxide (N_2_O) are the most important gases responsible for climate change and global warming in terrestrial ecosystems [2,3]. The high spatial and temporal variability of plant and microbial processes associated with the production and consumption of GHGs on agricultural lands is a major uncertainty in both global emission estimates and local effects within specific production systems [4]. Field-based quantification of these gases that incorporate the local environmental conditions, management practices, and crop types can be extrapolated to provide important regional data sets on the long-term impacts and sustainability of renewable biofuel systems.

Tropical perennial grasses such as sugarcane and napiergrass (*Pennisetum purpureum* Schumach.) are under consideration for bioenergy production due to their high productivity and physiological characteristics that limit photorespiration and increase nutrient and water use efficiency [5–7]. Sugarcane is a high-yielding, perennial grass of South Pacific origin that is well known for supporting a drought resistant robust root system that can improve soil structure and accumulate C on marginal lands [8–10]. Recent estimates by the Food and Agriculture Organization (FAO) have reported that over 22 million hectares of the world’s agricultural lands are dedicated to sugarcane production. Brazil, the largest sugarcane producing country, allocates about 45% of its 8 million ha croplands to ethanol production [10,11]. Tropical sugarcane dry biomass yields may range from 25.9 Mg ha^-1^ yr^-1^ in Brazil to 40 Mg ha^-1^ year^-1^ in Hawaii [12,13]. Napiergrass, another African origin warm-season perennial grass has been found to produce more than 45 Mg ha^-1^ year^-1^ in Florida and, similarly, between 40 and 53 Mg ha^-1^ year^-1^ in Hawaii [7,14,15]. However, under optimal conditions dry matter yields as high as 88 Mg ha^-1^ year^-1^have been recorded in El Salvador [7,16].

Sugarcane and napiergrass can maintain high biomass yields when managed as zero-tillage, ratoon harvest systems. The ratoon harvest practice, which cuts the biomass near the surface of the soil without disturbing the belowground root system to allow rapid vegetative regrowth, is central to the net GHG balance of these systems due to no or reduced field-based operational GHG emissions, decreased net GHG flux as a result of reduced soil disturbance or loss, and increased belowground soil organic carbon (SOC) storage [1,7,9,16,17]. Tropical C4 grasses are known to have the largest root biomass among agricultural crops and hence have the potential to influence the flow of C and GHG flux in biofuel feedstock production systems. Both sugarcane and napiergrass are water intensive species that have been shown to utilize available water and nutrients by expanding their root systems during their growth cycles and following harvest events [7,8,18]. Root biomass and plant residues have a direct effect on GHG emissions from the soil surface; the respiration of live roots and mycorrhizae contributes to CO_2_ efflux. Whereas, additional CO_2_, N_2_O and CH_4_ are produced through the microbial decomposition of dead roots and other organic matter in the rhizosphere. If gross primary productivity, partitioning of fixed C belowground, and the C use efficiency of the soil microbial community are high, then soil C accumulation can be rapid.

Designing sustainable sugarcane and napiergrass feedstock production systems for Hawaii requires accurate information on their performance under water limited conditions, potential SOC storage, and GHG emissions. Given the important contribution of roots to SOC, there also is a need for reliable estimates of root biomass and root distribution down the soil profile. An accurate accounting of total root C sources is critical for assessing the overall plant-derived C inputs into the soil [19]. Sumiyoshi et al. (2016) recently reported the critical role of root inputs and decomposition to building SOC in ratooned perennial grass systems on Oahu, yet there remains a lack of data that can be used to fully understand the role and contribution of root biomass to SOC in C4 cultivated grass systems across the tropics [20,21].

Globally, water use and sustainable intensification of feedstock production through crop and management choices are two key issues of particular relevance when considering the environmental impacts of a biofuel production system. To address these issues, the objectives of this study were (i) to quantify and compare GHG fluxes under two potential biofuel feedstocks: conventional two-year cycle sugarcane and ratoon harvested (every 6 months) napiergrass, (ii) to compare sugarcane and napiergrass aboveground biomass, and quantify their respective belowground root biomass and distribution down the soil profile, and (iii) to assess short-term changes in SOC. The evaluations were conducted at two irrigation treatments: 100%, and 50% of current commercial practice.

## Materials and Methods

### Study site and experimental design

The field experiment was located in the central isthmus of the island of Maui, Hawaii (20.89°N, 156.41°W) on Hawaiian Commercial and Sugar (HC&S) lands, the only remaining sugarcane plantation in Hawaii at the time of the study. The study was carried out on private land; we confirm that the owner of the land, Alexander & Baldwin, Inc., gave permission to conduct the study on this site. Currently (in 2016), HC&S is transitioning from conventional sugarcane production to diversified agriculture to include some combination of pasture, forage production, bioenergy feedstock, and an agricultural park. The experimental plots were installed in 2011 on a highly weathered, very-fine, kaolinitic, isohyperthermic Typic Eutrotorrox of the Molokai series. This soil is well drained, rocky, and has deep, well-defined horizons below the plow layer [22]. Annual air temperature and precipitation for the experimental site were 23.4°C and 241 mm during the study period, which are consistent with long-term averages for the area [23]. The elevation of the commercial field is 100 meters above sea level and has an area of 72 hectares.

The full experiment was designed as a strip-plot, group-balanced design with two factors, irrigation and species with three replicates (blocks). Irrigation was applied at the standard plantation rate (100%), and two deficit irrigation rates (75% and 50% of plantation standard). The original trial included four species, sugarcane, energycane (*Saccharum officinarum x Saccharum spontaneum*), napiergrass, and sweet sorghum (*Sorghum bicolor* (L.) Moench). Irrigation level was applied uniformly down a row of plots planted along a set of buried irrigation lines. Within those lines, species were assigned randomly to plots in an orthogonal design for the three blocks. For this study, two crops (sugarcane and napiergrass) were evaluated at two irrigation levels (50% and 100%). From November 2011—October 2012, 1,245 mm water ha^-1^ were applied to the 100% plots and 633 mm water ha^-1^ were applied to the 50% plots, for an actual deficit treatment of 50.8%.

Field plots were established on June 26, 2011 in a recently harvested sugarcane field that had been in a cane-on-cane rotation for over 100 years. Each subplot had an area of 67.1 m^2^. The sugarcane plots were planted with seed cane, variety HA65-7052 supplied by HC&S. The napiergrass seed crop was supplied from a harvested population at the University of Hawaii’s research station in Waimanalo, Oahu. To control weeds, a pre-emergence herbicide mix containing atrazine (1-Chloro-3-ethylamino-5-isopropylamino-2, 4, 6-triazine), 2, 4-D (2, 4-Dichlorophenoxyacetic acid), Prowl ((N-1- ethylpropyl)-3, 4-dimethyl-2, 6 dinitrobenzenamine), Rifle (3, 6-dichloro-2-methoxybenzoic acid), and Velpar (3-cyclohexyl- 6-dimethylamino-1-methyl-1, 3, 5-triazine-2, 4(1H,3H)-dione) was applied once three weeks after planting. Each plot received a total of 345 kg N ha^-1^ (as liquid urea: 46-0-0) applied through the drip irrigation system monthly once the crops were established and concluded after 10 months. The timing and rate of urea application were optimized for the two-year sugarcane crop and were based on current HC&S plantation practices. The napiergrass plots received the same amount of fertilizer as the sugarcane plots. Deficit irrigation treatments were postponed during all fertilizer application events.

Due to an initial crop failure caused by insect damage, the napiergrass plots were replanted on September 16, 2011, 87 days after the initial planting. To ensure initial germination and survival, irrigation was applied weekly until all of the plots were established. Deficit irrigation treatments were then applied to the field from November 13, 2011. The napiergrass plots were ratoon harvested four times during the study period; at 6 months on March 13, 2012, at approximately 12 months on September 25, 2012, at 18 months on March, 13, 2013, and finally on May 15, 2013 when the surrounding commercial sugarcane field was harvested.

### Environmental measurements

Two weather stations (HOBO logger model H-21, Onset Computer, Bourne, MA, USA) were installed in the experimental field. Each station recorded hourly measurements of precipitation, solar radiation, wind speed, relative humidity and air temperature. In addition, soil temperature and moisture were collected concurrently with the flux measurements using a Stevens Hydra Probe II soil sensor (Stevens Water Monitoring Systems, Inc.). Water filled pore space (WFPS) at a soil depth of 5 cm was calculated from soil moisture data collected by the Hydra Probe using the following equation:

$$WFPS\;\left(\%\right)=\frac{Vol\;\left(\%\right)}{1-\frac{\rho \;\left(g\;cm^{-3}\right)}{2.94\;\left(g\;cm^{-3}\right)}}$$ (1)

where *ρ* is bulk density specific to the field soil (1.35 g cm^-3^), *Vol* is volumetric water content, and 2.94 is the particle density of a similar Maui Oxisol soil [3,24].

### Gas flux measurements

Soil surface gas flux measurements were collected using custom static vented chambers as specified in the GRACEnet (Greenhouse gas Reduction through Agricultural Carbon Enhancement Network) protocol [25]. Each chamber was constructed out of polyvinylchloride (PVC) material (15.24 cm diameter x 15.5 cm tall) and included a permanently installed collar buried to a depth of 8 cm and a fitted styrene cap used only during sampling events. Caps were designed to limit leakage and minimize disturbance associated with sample removal. A total of six collars were installed within each experimental plot; three within the row, and three within the inter-row. Installation occurred on September 26, 2011 and collars were allowed to settle for 23 days prior to the first sampling date. Samples were collected by sealing each chamber and using a 10 mL polypropylene syringe and extracting 8 mL of headspace air through a septum on the styrene lid at 0, 15, 30, 45, and 60 minutes after chamber closure. Each gas sample was injected into an evacuated Exetainer® (Labco Limited, UK) fitted with a Doubled Wadded Teflon/Silicon septa (Labco Limited, UK) for short-term storage. Samples were analyzed using a Shimadzu GC-2014 Gas Chromatograph (Shimadzu Scientific Instruments, Inc.). Flux rates were calculated by assuming a linear change in gas concentration over time [26,27]. Row and inter-row flux measurements were averaged together to determine representative plot treatment means for each species [25,28].

Mid-morning flux measurements were collected at least once a month from October 20, 2011 to October 5, 2012. In addition to the monthly flux measurements, samples were collected consecutively for 8 days following a fertilizer application event on April 27, 2012 and for a 5-day interval for 30 days following napiergrass harvest events on March 15, 2012.

### Global warming potential

All GHGs were assigned a global warming potential (GWP) value based on their radiative efficiency relative to that of CO_2_ over a 100 yr^-1^ time scale as established by the IPCC (2007): when the GWP of CO_2_ = 1, then the GWP for N_2_O and CH_4_ are 298 and 25 respectively [3,4,20,27]. To assess the overall impact of N_2_O and CH_4_ on the GHG budget from these two crops, their flux values were converted into CO_2_ equivalents by multiplying the cumulative flux of each gas on an annualized basis by its GWP ratio; these values were then totaled for each species and irrigation treatment level as described by Smith *et al*.[27]. For many agricultural systems, the difference between net C uptake by plants and losses of C from crop harvest and from the microbial oxidation of crop residues and soil organic matter are reflected predominantly in changes in soil organic C [29]. Therefore, in net GWP accounting, net CO_2_ flux is calculated on the basis of the change in soil C stock and CO_2_ costs of the agronomic inputs [29–31].

### Baseline soil sampling

Initial soil sampling was conducted in June of 2011. Soil cores were collected in 20-cm depth increments up to a vertical depth of 2.4 m. Cores were extracted using a standard wet core diamond tipped drill bit with an internal diameter of 7cm (Diamond Products Core Borer, Elyria, Ohio, USA). Each core barrel was inserted into the soil by a rotating hydraulic drill to minimize compaction within the barrel and to ensure accurate depth measurements. Soil samples were frozen at field moisture conditions until laboratory analysis. Soil samples were sieved at <2 mm and dried for 48 hours at 105°C. Subsamples were ground to pass through a 250 micron sieve for heterogeneity, weighed, and analyzed for C and N concentration by combustion using a Costech ECS 4010 CNH Analyzer (Costech Analytical Technologies, Inc., Valencia, CA, USA).

### Root biomass sampling and soil carbon change

Sugarcane and napiergrass root biomass and distribution were determined at end of year 1 and year 2 using a destructive root sampling technique [7]. Three soil cores (65-mm inner diameter) were collected to a depth of 100 cm in 20 cm increments from the center plant row of each plot, and adjacent to a live clump of sugarcane or napiergrass. Soil samples were air dried, and sieved using a standard 2 mm sieve. To estimate root biomass in each sample, coarse live and dead roots greater than 2 mm were collected from the sieve surface and added to any remaining identifiable roots that were hand picked from the soil that passed through the sieve. All roots were dried at 65°C and weighed. Subsamples of soil (that passed through the 2 mm sieve) from the corresponding soil cores were oven dried, homogenized, weighed, and analyzed for a change in soil C concentration from the baseline soil data. Soil C stock was determined using the equivalent soil mass method [32].

### Statistical analysis

Differences in gas flux rates by species and irrigation level were analyzed statistically using MINITAB 16 (Minitab Inc., State College, PA). A significance level of p = 0.05 was established for all tests. Maximum likelihood repeated measures ANOVA using a compound symmetry covariance structure was used to determine these effects over time; where species and irrigation levels were considered fixed factors, replicate blocks were treated as random, and date of sampling was considered the repeated factor. Gas flux following harvest and the targeted fertilizer application events were analyzed separately in the same repeated measures ANOVA format. All N_2_O flux data was log transformed to correct for non-normality and severe skew. No other transformations on the flux data were necessary, and unless otherwise stated, basic ANOVA assumptions were met. Cumulative annual fluxes for GWP assessment were estimated using linear interpolation between sampling dates. A least-squares ANOVA was used to test the effect of species and irrigation levels on soil core root biomass and on above ground biomass. All root biomass data was log transformed to correct for non-normality and skew. When significant, Tukey-Kramer post-hoc analysis was used for comparison of treatment means for fluxes, soil C, and root biomass.

## Results and Discussion

### Monitored water, temperature, and soil GHG flux

**Fig 1:**
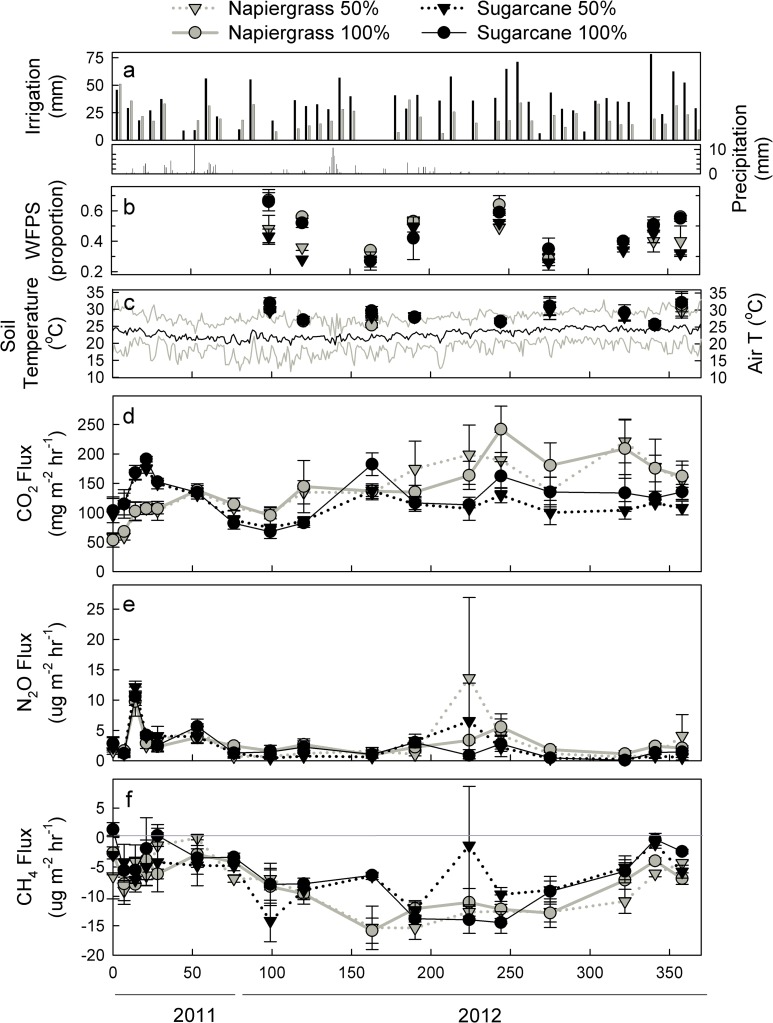
Time series for environmental variables and gas fluxes. Precipitation, irrigation (a), soil water filled pore space (b) and temperature (air and soil) (c), and greenhouse gas flux (d-f) for one production cycle of commercial field #609 at HC&S. Mean values (± one standard error) are shown for static chamber measurements of CO_2_ (d), N_2_O (e), and CH_4_ (f) flux.

Precipitation was low during the study period and irrigation provided most of the water inputs to the system (Fig 1A). The highest soil WFPS was recorded during peak irrigation events (Fig 1B). Although precipitation events were infrequent and generally resulted in minimal rainfall, a significant positive correlation was found between WFPS and precipitation (p ≤ 0.001; r^2^ = 0.14). Mean WFPS values ranged from 38% to 50% during the study period and did not differ between the species. Deficit irrigation effects on WFPS were dependent on date (p = 0.002), but for the majority of the sample dates WFPS was lower under the deficit treatment. Although the water deficit was 50%, the WFPS was reduced by only 4% and the soil moisture remained within the range for microbial activity associated with the production and consumption of GHG [33].

The range of soil temperatures found during this study was narrow compared to similar studies conducted in temperate environments [28,34,35]. Mean soil temperature for the study period was 28 ± 0.29°C and ranged from 24–36°C (Fig 1C). This range is consistent with the tropical geography, subtropical climate, limited seasonal variation in the saddle of Maui (± 5°C), and consistent elevation between the treatment plots [36]. Additionally, sampling periods were restricted to the hours between 0700 and 1100 to control for significant variation in diurnal temperatures. Soil temperature varied by date (p ≤ 0.0001) but did not differ between the species or irrigation treatment (Fig 1C).

Overall, soil CO_2_ flux was the greatest source of GHG in this study (Fig 1D). Consistent with the isohyperthermic, sub-tropical climate, seasonal patterns of CO_2_ flux were minimal during the study period. Soil CO_2_ emissions were positively correlated to air temperature (p < 0.001, r^2^ = 0.09) and total precipitation (p < 0.004, r^2^ = 0.08) but had no significant relationship to WFPS or soil temperature. The lack of relationship between soil temperature and CO_2_ emissions, even as one existed between air temperature and CO_2_ emissions, suggests a direct control of root (autotrophic) respiration on net CO_2_ flux. Air temperature was not significantly correlated to soil temperature, suggesting a strong buffering effect of dense vegetation on the air-soil interface. The subtropical climate and irrigation schedule may have provided optimal soil temperature and moisture conditions for a baseline microbial (heterotrophic) respiration rate throughout the study. Given that there was no correlation between soil temperature and respiration, heterotrophic respiration may have remained constant while autotrophic respiration increased with air temperature as the plant itself was the direct mechanistic connection between canopy air temperature and soil respiration. Reduced irrigation resulted in lower CO_2_ emissions compared to the 100% plots (p = 0.09). With the exception of one month, sugarcane plots exhibited significantly lower CO_2_ emission than the napiergrass (p = 0.06, for the time and species interaction). Mean fluxes were 156 ± 6.8 mg CO_2_ m^-2^ hr^-1^ for napiergrass and 112 ± 4.1mg CO_2_ m^-2^ hr^-1^ for sugarcane.

CO_2_ flux in this study was lower than fluxes reported for conventionally managed sugarcane on an Oxisol in Brazil [37] and for other perennial grasses. For example, values reported for *Miscanthus* production in England averaged around 230 mg CO_2_ m^-2^ hr^-1^[34]. There have been no published values for CO_2_ emissions under napiergrass to date. Soil temperature was not a significant factor in the production of CO_2_ in this study, which was not surprising considering the limited range in average temperatures during the sampling period. Diurnal variation in CO_2_ emissions has been well studied and has been found strongly linked to air and soil temperatures but due to the tropical nature of our system and relatively small differences in day and night temperatures large variations were not expected. In light of this expectation, this study focused on mid-morning emissions, which limited the influence of extremes in temperatures and represented the average daily condition [38].

Monthly fluxes of N_2_O were low and constant throughout the sampling period; no clear seasonal trends in N_2_O emissions were present (Fig 1E). There was a significant positive correlation between soil N_2_O emissions and soil WFPS (p < 0.0001), but no relationship between N_2_O flux and soil temperature. A significant spike in N_2_O flux occurred in April (p = 0.022 for time effect) coinciding with a fertilization event. Matson *et al*. (1996) reported similar, very low N_2_O flux from commercial sugarcane fields on Maui with short-lived spikes in flux following fertilization (see next section for a more detailed fertilization effect discussion). Fluxes from napiergrass were approximately 70% higher than sugarcane; mean N_2_O flux was 2.49 ± 0.59 μg N_2_O m^-2^ hr^-1^ for napiergrass and 1.46 ± 0.29 μg N_2_O m^-2^ hr^-1^ for sugarcane (p = 0.047). There was no significant irrigation effect on the rate of N_2_O emissions from these plots on a monthly basis.

Flux of N_2_O in this study was consistent with that from similar perennial bioenergy systems [3,34,39]. Nitrous oxide flux was significantly influenced by WFPS, which is consistent with other studies that found high soil moisture responsible for N_2_O production due to increased rates of nitrification and denitrification [39]. The actual pathway for producing N_2_O in these soils is challenging to discern. Likely, N_2_O is a result of both of these processes within the range of WFPS that dominates in these soils.

Methane uptake, or oxidation, was the predominant CH_4_ process during this study (Fig 1F). Irrigation had no effect on CH_4_ flux but there was a significant species and time interaction (p = 0.001). The lowest CH_4_ uptake rates were measured on the napiergrass plots 15 days after harvest (-22.71 μg m^-2^ hr^-1^ for 50% and -19.64 μg m^-2^ hr^-1^ for 100%); a significant increase in oxidation was noted during this time (P = 0.008). Sugarcane had similarly large negative fluxes (-19.72 and -18.57 μg m^-2^ hr^-1^) but these occurred in January and could not be explained by a disturbance or harvest event for this crop.

A negative flux (or increasing uptake) in upland agricultural systems generally indicates that methane oxidation from the atmosphere is taking place in these soils [33,34]. There have been very few site-specific or regional datasets collected on CH_4_ flux in sugarcane and none on napiergrass. But, methane oxidation has been reported in several perennial grass systems with comparable rates to ones found in this study: -2.5 μg m^-2^ hr^-1^ for *Miscanthus* in NE England [34], -6.0 to -2.0 μg m^-2^ hr^-1^ for *Miscanthus* in SW Germany [3] and 0 to -1.14 μg m^-2^ s^-1^ [39] and 1 μg m^-2^ s^- 1^ for sugarcane in Queensland, Australia [40].

### Targeted GHG flux after harvest and fertilization

**Fig 2:**
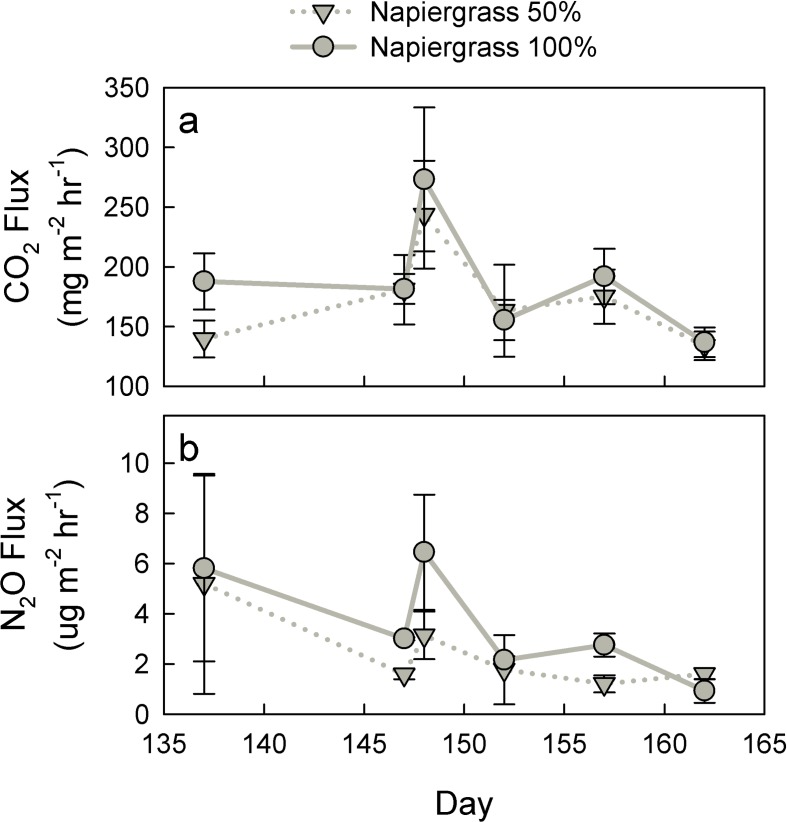
Gas fluxes peak following harvest. CO_2_ (a) and N_2_O (b) flux following harvest; values are means (± one standard error).

During ratoon harvest, a net increase in soil CO_2_ flux was expected due to CO_2_ losses through root turnover and disruption of the rhizosphere that were greater in magnitude than the decrease due to reduced plant activity. Further, because available water promotes nitrification and associated gaseous losses, it was expected that the soil N_2_O losses would be reduced from the deficit irrigation compared to the full, 100% irrigation level. Intensive gas flux sampling during the 15 days following a March 2012 harvest event tested this hypothesis. Within a day of the napiergrass harvest, soil CO_2_ emissions rapidly increased by 35% for the deficit irrigation and by 51% for the 100% irrigation treatments to means of 243.6 ± 45.1 mg CO_2_ m^-2^ hr^-1^ and 273.2 ± 60.3 mg CO_2_ m^-2^ hr^-1^ respectively (Fig 2A). Thereafter, soil emissions dropped to 133.8 ± 11.9 mg CO_2_ m^-2^ hr^-1^ for the 50% treatment and 136.8 ± 12.5 mg CO_2_ m^-2^ hr^-1^ for the 100% after 15 days.

The unexpected overall reduction in CO_2_ flux for napiergrass in the weeks following harvest, in fact, likely is responsible for the only date reported in the previous section (Fig 1D) where monthly sugarcane CO_2_ emissions were greater than napiergrass. A reduction in N_2_O flux from the 50% irrigation compared to the 100% irrigation occurred only the first day post-harvest (p = 0.009 for the time and species interaction) (Fig 2B). The disturbance effect of harvest was a short-term reversal of the long-term trends in flux, and is likely to have minimal impact overall on the GHG balance of the system.

CO_2_ flux rates increased substantially following harvest of the napiergrass plots. Although short lived, this significant increase may have been an immediate result of root response to a shift in aboveground plant physiology. A similar increase in CO_2_ production following harvest was found in *Miscanthus* grown in Germany [3] where an increase of approximately 300 mg m^-2^ hr^-1^ was measured after harvest but returned to previous emission levels within one week. Harvest frequency could have a significant effect on the overall soil CO_2_ emissions if root respiration was increased during these events or if root dieback occurred, which would stimulate decomposition.

**Fig 3:**
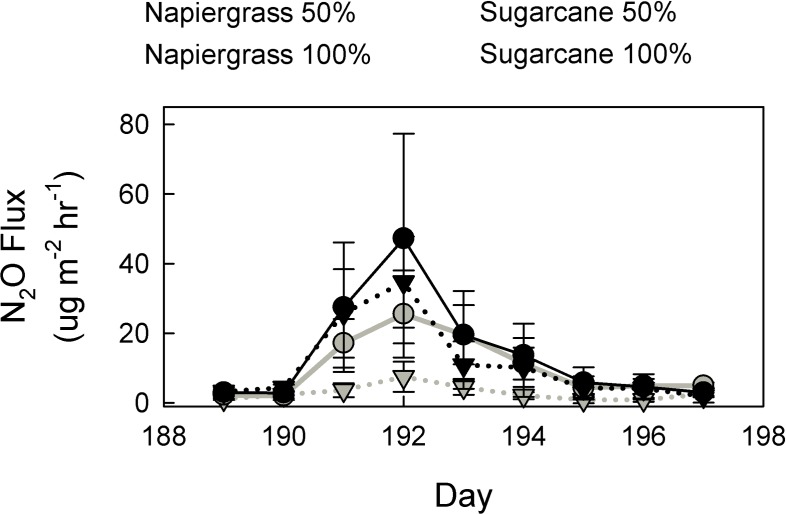
N_2_O flux increases after fertilization. N_2_O flux following fertilization; values are means (± one standard error).

In many agricultural systems, inefficient water and fertilizer application result in substantial gaseous losses of N_2_O [4,41,42], thus it was hypothesized that N_2_O flux would increase following fertilization for both species, but less so under deficit irrigation. This hypothesis was tested by intensive daily measurements of N_2_O flux for nine days following the application of fertilizer through the buried drip irrigation lines in April 2012. In contrast to the low monthly means, large N_2_O emissions were measured following fertilization (Fig 3). For both species, emissions rose within 24 hours of fertilizer application and rapidly peaked on the 3^rd^ day with average rates of 15.87 and 45.95 μg N_2_O m^-2^ hr^-1^ for napiergrass 50% and 100% respectively and 55.45 and 103.96 μg N_2_O m^-2^ hr^-1^ respectively for sugarcane. Emissions returned to pre-fertilization levels by day six. Sugarcane plots emitted more than double the amount of N_2_O during this event than the napiergrass and emissions were significantly greater under the 100% treatment (p = 0.02 for species and p = 0.09 for treatment). Mean emissions were approximately 6.51 ± 1.27 and 13.57 ± 2.80 μg N_2_O m^-2^ hr^-1^ for napiergrass and sugarcane respectively over the nine days.

On average, soil N_2_O flux was greater for napiergrass than sugarcane, except immediately following fertilization (Fig 1E). After fertilizer application, a short-lived but measureable peak in N_2_O occurred for the sugarcane (and not napiergrass) (Fig 3). This short-lived spike caused a 100% increase in emissions from the previous day. Even though the high fluxes did not last for more than 24 hours, the magnitude of this increase is exacerbated due to the relatively small fluxes found during the rest of the year. In general, losses of N_2_O from these soils were low during the study period and ranged from ~0 to 10 μg m^-2^ hr^-1^ when targeted harvest and fertilizer events were not included.

### Greenhouse gas balance

Soil N_2_O and CH_4_ fluxes were much lower in magnitude than CO_2_ during this study, but their potential to trap infrared radiation and offset the overall balance between these gases is much greater. In order to compare the impacts of these cropping systems and treatment effects on the net emissions to the ecosystem, N_2_O and CH_4_ were converted to CO_2_ equivalents (CO_2eq_) based on the IPCC 100-year projections. No statistically significant differences in total CO_2eq_ were found between the species or irrigation and the mean annual CO_2 eq_ emissions were 9.05 ± 3.40 Mg ha^-1^ yr^-1^. For the individual gases: The annualized data for CH_4_ found napiergrass to have higher rates of consumption than sugarcane at both irrigation treatment levels. Highest rates were found under the Napier 100% treatment -0.149 ± 0.002 Mg CO_2eq_ ha^-1^ yr^-1^ and the lowest -0.072 ± 0.003 Mg CO_2eq_ ha^-1^ yr^-1^ were found under sugarcane 50% irrigation. The GWP of N_2_O was also highest under the Napier crop at 100% irrigation (0.227 ± 0.002 Mg CO_2eq_ ha^-1^ yr^-1^) and lowest under sugarcane at 50% irrigation (0.116 ± 0.004 Mg CO_2eq_ ha^-1^ yr^-1^). The net CO_2_ equivalents between CH_4_ and N_2_O was positive for each system but were found to be lowest in sugarcane at the 50% irrigation treatment (0.044 Mg CO_2eq_ ha^-1^ yr^-1^).

Replacing fossil fuel with biofuel is only environmentally viable if the net GHG footprint of the production system is reduced. One component of that system-level balance is the effect of replacing annual arable crops with perennial bioenergy feedstocks on net GHG production. Multiple recent studies have shown that temperate perennial bioenergy crops only reduce measured GHG emission compared to annuals if they are not fertilized [34,43,44]. In Brazil, maintaining N amendment rates and improving nitrogen use efficiency (NUE) through genetic improvement and better management practices are critical to increasing biofuel production sustainably [45]. In these Brazilian systems, N losses to leaching and N_2_O emission can be as high as 5.6% of added N. In this study, gaseous losses were consistently very low because of efficient fertilizer and irrigation practices in use on the plantation. Buried irrigation lines through which the fertilizer was applied could be responsible for mitigating large losses of N_2_O in these soils by targeting nutrient addition to root zone and minimizing surface-based losses [46]. During the intensive measurements immediately following fertilization, approximately 0.1% of added N to the system was lost under the napiergrass compared to approximately 0.2% under the sugarcane (Fig 3). On an annualized basis, out of the 345 kg N ha^-1^ added to the experimental plots in the first year, approximately 6.79 kg N in N_2_O ha^-1^ yr^-1^ was lost to the atmosphere from the soil, which is around 2% of the total N applied to the whole system. This value is considerable lower than the 3–5% losses generally expected from agricultural sites [4,41]. This N balance suggests that during fertilization, the plantation is adequately managing their water and crop resources to minimize N lost from the system.

There have been several recent studies that suggest napiergrass has the capacity to fix biologically available nitrogen (BNF) in soils that receive no fertilizer inputs [47,48]. In de Morais *et al*. (2012), napiergrass accessions were able to obtain between 36 to 132 kg N ha^-1^ yr^-1^ from BNF on an agricultural Ultisol in Brazil. The potential to exploit this crop characteristic and minimize fertilizer inputs to these systems should be explored further to address the long-term sustainability of biofuel agriculture especially, if napiergrass is chosen as a feedstock.

### Yield and root biomass

Dry biomass yields for the 100% irrigation treatments were comparable to similar studies in which ample rates of nutrients and irrigation were applied; e.g., 45 Mg ha^-1^ for napiergrass in Florida [49] and 86 Mg ha^-1^ year^-1^ for sugarcane in Brazil [13]. The highest yielding plots at 100% irrigation after the 2-yr crop cycle were sugarcane (73.9 ± 10.2 Mg ha^-1^) followed by napiergrass (47.7 ± 5.8 Mg ha^-1^). However, a high coefficient of variation in one of the replicate sugarcane plots resulted in these differences being statistically non-significant. In all plots, the 100% treatments accounted for significantly greater total yields after 2-years of growth (p = 0.012). The 50% deficit irrigation treatment caused a 60% reduction in yield for sugarcane (to 29.4 ± 3.9 Mg ha^-1^) and a 31% reduction in yield for napiergrass (to 32.9 ± 4.6 Mg ha^-1^).

**Fig 4:**
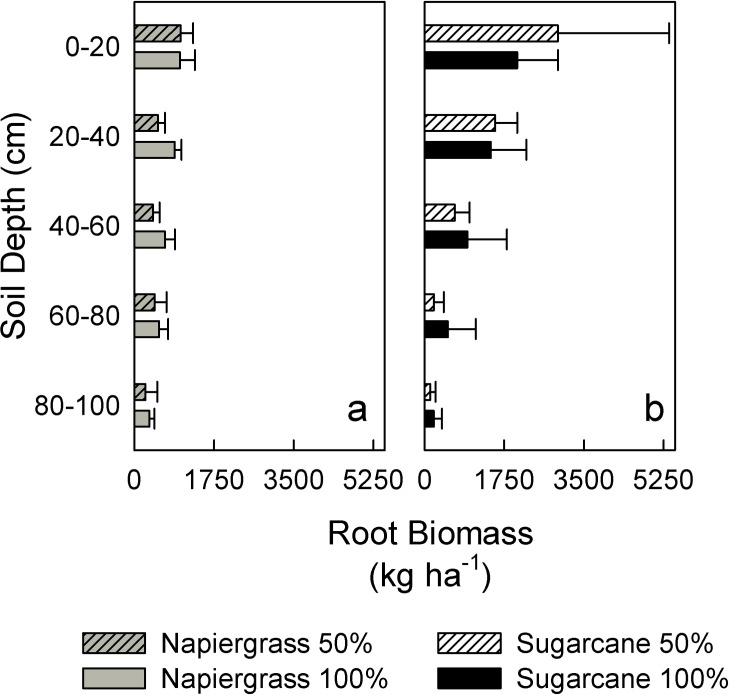
Species-specific root biomass and depth distribution. Total root biomass (year 1 plus year 2) by depth for napiergrass (a) and sugarcane (b); values are means (± one standard error).

Species did not affect root biomass, but deficit irrigation decreased roots for both sugarcane and napiergrass in the first year (p = 0.003) (Table 1). Root biomass in both species declined from the first to second year (p = 0.0001). The majority of root biomass was located within the surface layers of the soil profile and decreased with depth (Fig 4). In year one, more than 74% of root biomass for both species was in the top 40-cm. In year two, these estimates declined to 56% for napiergrass but increased to 77% for sugarcane. Other studies have reported similar patterns with depth for both napiergrass [7,49] and sugarcane [8,50,51]. The buried drip irrigation system and fertigation (i.e., injection of soluble fertilizer through the irrigation lines) at HC&S likely reduced the need for an extensive deep root system. During root sieving, observational differences were noted between the two species; sugarcane had more coarse roots in the surface depths whereas the napiergrass seemed to produce large amounts of very fine roots that were under-represented in the mass-balance approach. These differences could be attributed to the morphological differences between the species or to rooting response of napiergrass following a harvest.

**Table 1:** Total stocks in above and belowground pools.

		Napiergrass		Sugarcane	
(Mg ha^-1^)		50%	100%	50%	100%
Yield	Yr 1	21.45 ± 3.90	33.06 ± 3.75	n.a.	n.a.
	Yr 2	11.45 ± 1.20	14.60 ± 2.06	29.39 ± 3.91	73.92 ± 10.20
	**Total**	**32.90 ± 4.63**	**47.66 ± 5.77**	**29.39 ± 3.91**	**73.92 ± 10.20**
Root biomass	Yr 1	4.77 ± 0.25	8.72 ± 1.60	5.48 ± 1.13	7.79 ± 1.08
	Yr 2	2.57 ± 0.40	3.44 ± 0.17	5.49 ± 0.99	5.16 ± 2.97
Δ Soil C stock	BL-yr 1	42.08 ± 6.98	31.03 ± 7.25	19.00 ± 10.07	25.70 ± 1.32
	Yr 1-yr 2	-7.36 ± 1.45	-1.89 ± 3.08	15.44 ± 12.32	0.97 ± 7.28
	**BL-yr 2**	**34.71 ± 6.27**	**29.14 ± 5.36**	**34.43 ± 19.04**	**26.67 ± 7.97**

Crop yield, root biomass, and soil C change: baseline to year 1, year 1 to year 2, and total over two years. Values are means **±** one standard error.

The 50% deficit irrigation treatment caused a 13% decrease in root biomass for sugarcane and a 38% decrease in napiergrass; this pattern was consistent at all depths (Fig 4). These modest reductions compared to the greater declines observed in the aboveground yield suggest greater allocation of resources belowground under deficit irrigation. Perennial grasses produce lengthy root structures that support plant growth and function during times of drought and the root response may have extended even deeper than our sampling to 1m [7,52]. Genotype-specific root distribution of *Miscanthus* influenced soil C sequestration over 14 years at the Rothamsted Farm in England as part of the European *Miscanthus* Improvement Project, reinforcing the importance of making system-specific estimates of root distribution and turnover [53].

### Soil carbon

**Fig 5:**
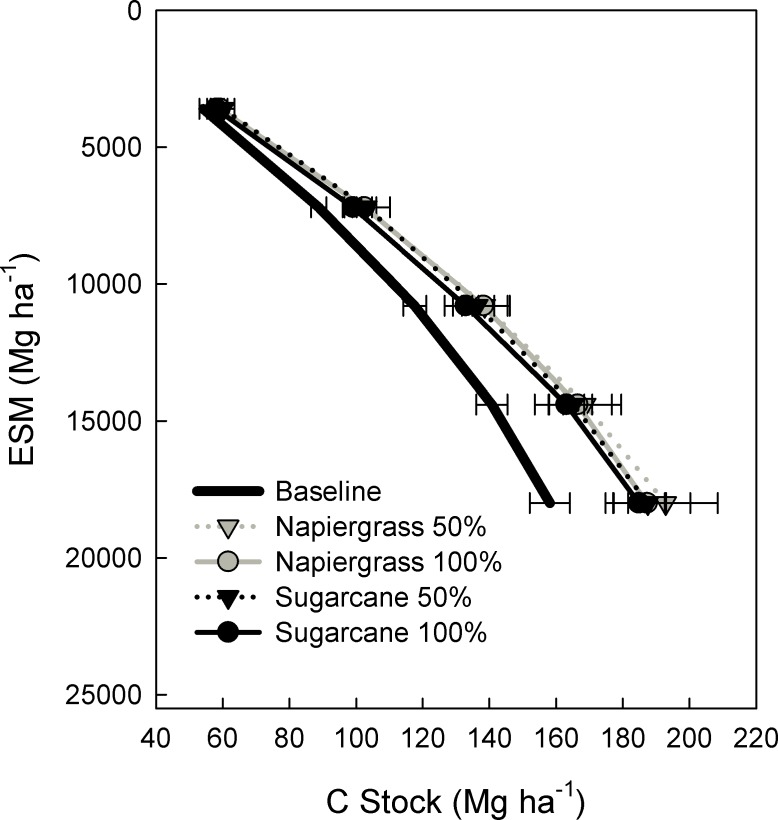
Two years of soil carbon accumulation. Soil C stock at baseline versus year 2 for sugarcane and napiergrass at 100% and 50% of the plantation irrigation level. Values are means (± one standard error); n = 10 for the baseline soils, n = 3 for the trial plots.

The baseline soil C stock in the equivalent soil mass of 18,000 Mg ha^-1^ (that occurred in the top 1.0–1.4 m of soil), determined from the mean of ten soil cores taken *a priori* from the planned field site, was 158.1 ± 6.9 Mg C ha^-1^. Soil C stock increased substantially from the baseline in the two-year period following cultivation in sugarcane and ratoon harvested, zero-tillage napiergrass (Fig 5). In just the first year, the total increase was 12.0% and 16.3% for sugarcane (50% and 100% irrigation respectively) and 26.6% and 19.6% for napiergrass (50% and 100% irrigation respectively). Soil C stock continued to increase from yr-1 to yr-2 in sugarcane but declined somewhat in napiergrass (Table 1) as equilibrium in the root system and associated rhizosphere and carbon inputs was reached following multiple ratoon harvests. The net percent increase over two years was 21.8% and 16.9% for sugarcane (50% and 100% irrigation respectively) and 22.0% and 18.4% for napiergrass (50% and 100% irrigation respectively). The mean annual increase in soil C stock over the first two years was 17.2 ± 9.5 and 13.3 ± 4.0 Mg C ha^-1^ yr^-1^ for sugarcane (50% and 100% irrigation respectively) and 17.4 ± 3.1 and 14.6 ± 2.7 Mg C ha^-1^ yr^-1^ for napiergrass (50% and 100% irrigation respectively).

Tropical ecosystems are capable of sustaining high rates of plant inputs and soil organic matter turnover, but also can sequester up to twice as much soil C when compared to temperate environments under zero-tillage [54]. The measured annual gains in SOC after the two years of the study is greater than similar studies in shallower soils of a temperate system (i.e., 0.10–1.0 Mg C ha^-1^ yr^-1^ in the top 30 cm [9]) and a tropical system with a fallow grassland starting point (i.e., up to 3.87 Mg C ha^-1^ in 30cm [21]). Similarly, soil C stock in the top 50-cm increased at a rate of 5 Mg C ha^-1^ yr^-1^ over five years of measurement in Texas under energy sorghum, which is not a zero-tillage system, cultivated on former, intensively-managed cotton lands [55]. Increasingly, evidence suggests that deep soil C dynamics play a critical role in driving total C stocks and response to perturbation such as land use and climate change [56,57]. Soils in the tropics and sub-tropics often are deep and rich in Fe-oxides clays that promote soil aggregation and associated beneficial physical properties that help support high productivity in agroecosystems. The greater depth of our soil C stock measurements alone accounts for some of the difference in magnitude between our results and many others. Sugarcane and napiergrass varieties can be bred to select for robust root systems capable of establishing and maintaining an extensive rhizosphere and growing very deep in search of water. The napiergrass variety was chosen for this trial based on early indicators of the potential for high aboveground biomass and dense, deep rooting system. Selection of crops specifically for their high productivity and partitioning of C resources belowground [58] likely also contributed to the observed high soil C accumulation rates [21].

In some cases, priming of the deep soil system has led to net soil C losses when fresh organic matter inputs are introduced to the deep soil profile [59]. Further, de Graaff et al. (2014) [60] documented in switchgrass that priming losses were greater in surface soil than deep soil. However in this case, the initial state of the belowground system was highly degraded following over 100 years of intensive sugarcane cultivation that relied on burning, deep soil ripping, and chemical fertilizers as part of the practice. As a result, prevalent soil C accumulation occurred over two years from the baseline measurement in this study. Some indications of fluctuations in soil C stock, particularly in napiergrass, are present likely in response to reaching equilibrium in the rhizosphere as the root system develops in the first year and root death and turnover occurs with each 6-month harvest cycle. In a previous study of napiergrass in Hawaii, soil C accumulation following the conversion of a grassy field to ratoon management was driven by high root biomass inputs and rapid root decomposition [21]. Soil C gains often resulted from microbial byproducts and biomass accumulation [61,62], particularly if the soil has biological, chemical, and physical properties that promote organic matter stabilization [63]. Zero-tillage systems promote active rhizospheres with high microbial diversity [64] and if those communities have high C use efficiency [65] and promote soil aggregation, then non-linear relationship between inputs and soil C accumulation can occur.

In combination, high productivity, belowground partitioning, high C use efficiency, stabilization of organic matter and microbial byproducts in soil aggregates promoted by both zero-tillage management and inherent mineralogy, and high clay content could result in the observed high initial C accumulation rates of this system. High clay content and concentration of iron-oxides contribute to good soil physical properties and further protect C within aggregates and organo-mineral interactions. These factors, along with the high inputs of organic matter from roots and the associated rhizosphere to depth of up to 1.4m on these plots suggest that SOC stocks in this system, starting from a highly degraded state, potentially increase even over short time scales. Rapid accumulation in the early phases of a management change will attenuate over time. More annual measurements and simulation of soil C accumulation with empirical or process models can help project longer-term patterns in these continuous ratoon systems.

## Conclusions

Linkages between harvest frequency, fine root turnover, and SOC accumulation occur in perennial grass systems [9,21,62] and the potential for climate change mitigation in soil carbon sequestration is important for long-term sustainability of bioenergy feedstock production within a renewable energy system. Many agroecosystems lose SOC during initial land conversion, but our results demonstrate the potential to sequester SOC in both of the sugarcane and napiergrass feedstock scenarios if conservation management practices, such as ratoon harvests and reduced tillage operations, are implemented. The environmental sustainability of feedstock production depends on a combination of water, fertilizer, and harvest management to maximize crop yield while reducing losses of C and greenhouse gases from soils. Reducing irrigation by 50% resulted in the lowest GWP for both species, but the tradeoff between reduced yields and improved GHG production and SOC accumulation ultimately determines the long-term sustainability of these systems in Hawaii. Shifting from conventional sugarcane to ratoon-harvested napiergrass is likely to have multiple benefits including a diversified agricultural system, reduced irrigation water requirement, improved energy security, and more sustainable GWP of the landscape.
